# A Novel *Saccharomyces cerevisiae* FG Nucleoporin Mutant Collection for Use in Nuclear Pore Complex Functional Experiments

**DOI:** 10.1534/g3.115.023002

**Published:** 2015-10-30

**Authors:** Rebecca L. Adams, Laura J. Terry, Susan R. Wente

**Affiliations:** Department of Cell and Developmental Biology, Vanderbilt University School of Medicine, Nashville, Tennessee 37240-7935

**Keywords:** nuclear pore complex, FG nucleoporin, *S. cerevisiae*

## Abstract

FG nucleoporins (Nups) are the class of proteins that both generate the permeability barrier and mediate selective transport through the nuclear pore complex (NPC). The FG Nup family has 11 members in *Saccharomyces cerevisiae*, and the study of mutants lacking different FG domains has been instrumental in testing transport models. To continue analyzing the distinct functional roles of FG Nups *in vivo*, additional robust genetic tools are required. Here, we describe a novel collection of *S. cerevisiae* mutant strains in which the FG domains of different groups of Nups are absent (Δ) in the greatest number documented to date. Using this plasmid-based Δ*FG* strategy, we find that a GLFG domain-only pore is sufficient for viability. The resulting extensive plasmid and strain resources are available to the scientific community for future in-depth *in vivo* studies of NPC transport.

The nuclear pore complex (NPC) is the essential, conserved, selective portal for nucleocytoplasmic transport in eukaryotic cells. By controlling transport across the NPC and maintaining the separation of transcription and translation machinery, intricate levels of gene regulation are supported in both single and multicellular eukaryotic organisms (reviewed in Raices and D’Angelo 2012). The 60–120 MDa NPC complex is built from multiple copies of a conserved set of ∼30 nuclear pore proteins (nucleoporins, Nups; reviewed in Field *et al.* 2014). Nups are organized into subcomplexes that assemble to generate a transport channel across the nuclear envelope (NE) with nuclear basket and cytoplasmic filament structures extending from the NE (Figure 1). Diverse technologies have been used to enhance our understanding of how structural Nups interact to build the NPC scaffold (Alber *et al.* 2007; Field *et al.* 2014; Chug *et al.* 2015; Stuwe *et al.* 2015). However, despite extensive study using a variety of approaches, questions remain regarding how the NPC forms a barrier to nonspecific transport of large macromolecules (>40 kDa) while at the same time facilitating specific import and export of molecules against concentration gradients (Rout *et al.* 2000; Yamada *et al.* 2010; Hulsmann *et al.* 2012; Lim *et al.* 2015). Importantly, the combined use of *in vivo* and *in vitro* experimental approaches is critical to fully unravel the mechanisms for nuclear transport and to define discrete Nup functions in a cell.

**Figure 1:**
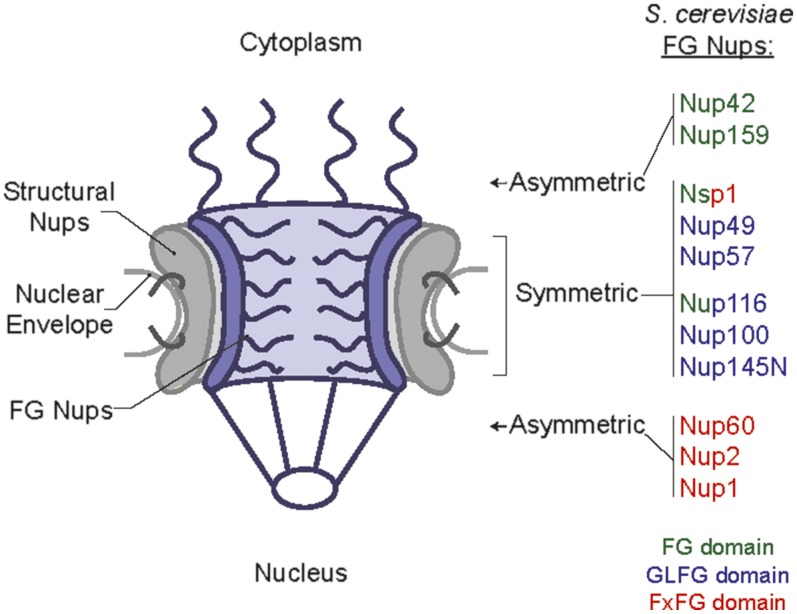
Schematic of NPC depicting relative structural location of FG Nups, based on Rout *et al.* (2000) with the image adapted from Adams and Wente (2013). FG Nups are color-coded based on the type of FG repeats enriched in their FG domains: Green, FG; Blue, GLFG; Red, FxFG. Nsp1 contains both FG and FxFG domains, and Nup116 contains both FG and GLFG domains.

The FG Nups (11 members in *Saccharomyces cerevisiae* and humans) are the class of NPC proteins that both generate the NPC permeability barrier and provide binding sites for facilitated transport (Hulsmann *et al.* 2012; Lord *et al.* 2015; reviewed in Terry and Wente 2009). Each FG Nup contains an unstructured domain with multiple phenylalanine-glycine (FG) repeat motifs separated by ∼10–20 spacer residues comprised mostly of polar amino acids. The 11 FG Nups are characterized by different types of FG repeat motifs (classified into FG; glycine-leucine-phenylalanine-glycine, GLFG; or phenylalanine-any-phenylalanine-glycine, FxFG domains; reviewed in Rout and Wente 1994). Unless specified, in this report, we use the terminology “FG” generically to refer to the entire family of FG, FxFG, and GLFG Nups or when referring to multiple FG domains in a subcomplex. In each FG Nup, structured region(s) flank the FG domain to allow interaction with scaffold Nups. These structural domains effectively anchor FG Nups at discrete NPC sites, either symmetrically in the channel or asymmetrically at the cytoplasmic or nuclear face (Figure 1; Rout *et al.* 2000). Furthermore, a trio of highly conserved symmetric FG Nups (in *S. cerevisiae*: Nsp1, Nup49, and Nup57; in vertebrates: Nup62, Nup58/Nup45, and Nup54) forms a subcomplex through interactions of their coiled-coil structural domains (Chug *et al.* 2015; Stuwe *et al.* 2015).

The unstructured FG domains are modeled to extend into the NPC transport channel (reviewed in Terry and Wente 2009; Kabachinski and Schwartz 2015). For facilitated movement through the NPC, specialized transport receptors bind both the cargo and the FG repeats of FG domains, allowing entry into and through the FG domain network (reviewed in Field *et al.* 2014; Kabachinski and Schwartz 2015). Directionality of transport is mediated by additional soluble factors found at the NPC faces, or in the nucleus or cytoplasm (reviewed in Kabachinski and Schwartz 2015). With regard to roles in inhibiting the diffusion of macromolecules, the vertebrate GLFG Nup98 is critically important (Hulsmann *et al.* 2012) and the *S. cerevisiae* orthologs Nup116 and Nup100 also contribute to the permeability barrier (Lord *et al.* 2015). Thus, due to their bifunctional role in inhibiting diffusion of molecules and providing binding sites for transport receptors, FG domains constitute the fundamental basis for selective nucleocytoplasmic trafficking.

To date, *S. cerevisiae* is an important model system for investigating FG domain function *in vivo*, and diverse mutant construction approaches have been developed over the nearly three decades of study. Analyses of strains generated with entire genes deleted found that some FG Nup encoding genes are individually essential (Hurt 1988; Davis and Fink 1990; Wente *et al.* 1992; Del Priore *et al.* 1997). Thus, for functional studies with full gene deletions, analysis is limited to nonessential genes. Early studies also used plasmid-based expression of *nup* FG domain deletion (Δ*FG*) alleles to complement lethal chromosomal *nup* null mutants, and demonstrated that most individual FG domains can be removed with no loss in cell viability (Nehrbass *et al.* 1990; Grandi *et al.* 1995; Iovine *et al.* 1995; Del Priore *et al.* 1997). Indeed, most plasmid-based individual Δ*FG* strains with only the FG domain absent have minimal growth and transport defects (reviewed in Terry and Wente 2009). Given such functional redundancy within the NPC, to analyze FG domain function, multiple combined deletions of sequences encoding different FG domains must be included within a given strain. However, with 11 FG Nups, the availability of auxotrophic markers to maintain multiple plasmids, each encoding individual *NUP* genes, has limited analysis using such a strategy to only a few Nups within one strain.

To overcome these limitations, we originally developed a collection of *S. cerevisiae* mutants wherein *NUP* genes, with only the sequence encoding the respective FG domain deleted (Δ*FG*), are expressed from the endogenous chromosomal locus (Strawn *et al.* 2004; Terry and Wente 2007). In this approach, Δ*FG* alleles were generated by replacement of the FG domain-encoding region of the *NUP* gene with a floxed *SpHIS5* “replacement” cassette, selection on media lacking histidine, and subsequent looping out of the *SpHIS5* sequence by expression of Cre recombinase (Strawn *et al.* 2004). The replacement cassette also included sequence encoding one of four small epitope tags (FLAG, myc, T7, or HA) that was retained with the remaining *loxP* sequence after *SpHIS5* was looped out. The resulting in-frame Δ*FG* gene expressed a protein with both the respective epitope tag and the translated *loxP* sequence, “TTLNITSYNVCYTKLL”, in place of the FG domain. By classic yeast genetic strategies, Δ*FG* alleles were then combined to generate higher-order, multiple Δ*FG* mutant strains (Strawn *et al.* 2004). Deletion of all five asymmetric FG domains results in a mutant strain with minimal growth and transport defects. Subsequent analysis went further to remove one or two symmetric FG domains from the background where all asymmetrical FG domains were deleted from Nup1, Nup2, Nup60, Nup42, and Nup159 together (Terry and Wente 2007). Functional analysis of such multiple, higher order Δ*FG* mutants for perturbations in the transport of different import and export cargos revealed that the absence of specific FG domains leads to unique transport defects (Terry and Wente 2007). Overall, FG domains serve specialized roles during transport, but it is unknown what attributes (FG type, spacer sequence, location within the NPC) lead to these particular functions.

Although the chromosomal Δ*FG* mutant strains have been instrumental in NPC functional analysis, they have several important caveats. First, chromosomal deletions preclude easy modification of genes in comparison to plasmid-based expression. Second, the remaining epitope and loxP tags result in nonspecific defects in some of the higher-order multiple Δ*FG* mutant strains. For instance, we previously reported that the lethality of *T7-loxP-nup1*Δ*FxFG myc-loxP-nup2*Δ*FxFG myc-loxP-nup60*Δ*FG HA-loxP-nup42*Δ*FG myc-loxP-nup159*Δ*FG T7-loxP-nup49*Δ*FG* is rescued by plasmid-based expression of untagged *nup49*Δ*FG* (Terry and Wente 2007). Therefore, our goal in this study was to generate a new collection of Δ*FG* mutants which (1) avoid indirect effects from epitope or loxP tagging during strain construction, (2) allow straightforward future mutational analysis of the sequences encoding individual domains, and (3) enable functional analysis of the resulting mutants.

We report here a new approach based on chromosomal null alleles complemented by plasmid-based expression of Δ*FG nups*, wherein each plasmid encodes multiple FG Nups that are colocated in specific NPC substructures. Using this strategy, we find that the FG domains of the Nsp1-Nup49-Nup57 subcomplex and those located exclusively at the nuclear (Nup1, Nup2, and Nup60) and cytoplasmic faces (Nup42, and Nup159) of the NPC can all be deleted without loss of viability. Although harboring severe growth defects, these deletions result in a new GLFG domain-only NPC. This collection will be of use to the community and set the stage for future experiments further probing of FG domain function *in vivo*.

## Results and Discussion

**Figure 2:**
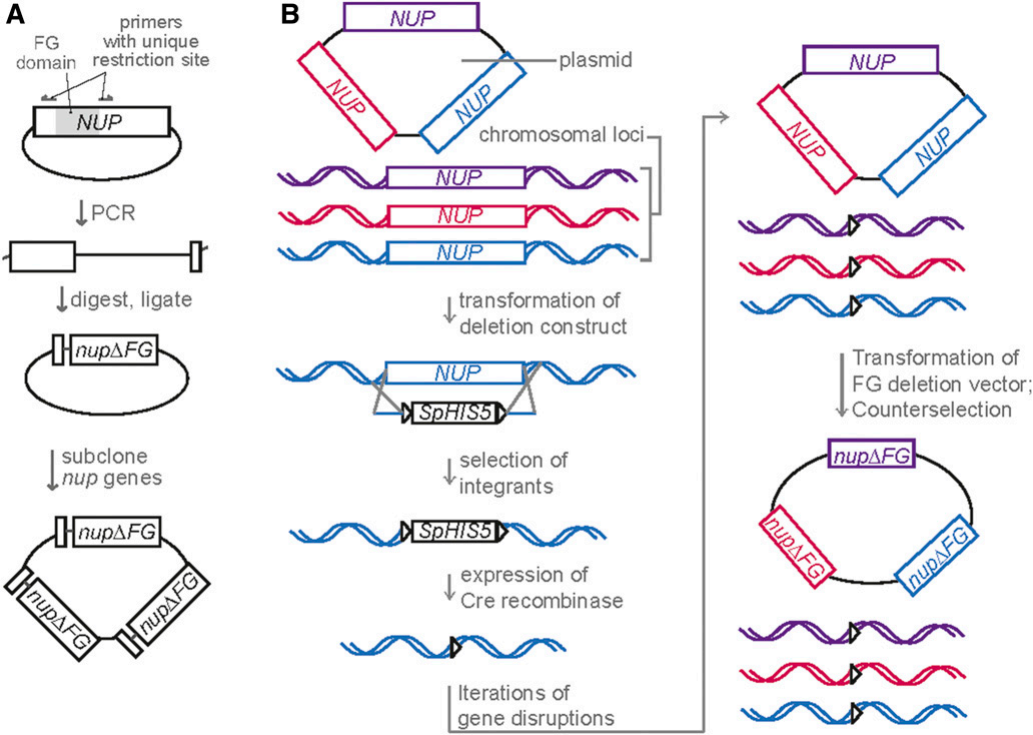
(A) Schematic of Δ*FG* plasmid construction. Centromeric plasmids encoding a *WT NUP* gene with its endogenous 5′ and 3′ UTR were PCR amplified with primers that annealed outside of the FG domain and generated a unique in-frame restriction site. PCR products were cut and ligated back together to generate the Δ*FG* plasmid. Δ*FG nups* or WT *NUPS* were subcloned into one plasmid encoding multiple genes (Table 2). (B) Schematic depicting Δ*FG* strain construction. Plasmids harboring multiple *NUP* genes were transformed into parent strains followed by disruption of the chromosomal ORF with sequence encoding floxed *Schizosaccharomyces pombe HIS5* (*SpHIS5*). *SpHIS5* was then looped out by transformation with a plasmid for inducible expression of Cre recombinase. Iterative transformation, disruption, and *SpHIS5* recycling cycles were used to generate indicated strains. Strains were subsequently transformed with Δ*FG nup* plasmids and counterselected.

Our efforts focused on deleting the FG domains of the conserved Nsp1-Nup49-Nup57 subcomplex in combination with deletion of FG domains from the nuclear (Nup1, Nup2, Nup60) and cytoplasmic (Nup159, Nup42) faces of the NPC. Analysis of such an octameric (eight) Δ*FG* mutant was not technically possible via former approaches. The basic strategy underlying the generation of a new collection of haploid *S. cerevisiae* Δ*FG* mutants included: (1) deletion of the entire endogenous *FG NUP* gene in the presence of a plasmid expressing the corresponding wild type (*WT*) *FG NUP*, and (2) shuffling the *WT FG NUP* plasmid for respective Δ*FG nup* constructs. Plasmids were engineered to allow expression of multiple *FG NUP* genes with their respective endogenous 5′ and 3′ UTRs (Figure 2A). Importantly, this plasmid-based expression strategy should not alter Nup stoichiometry within the NPC, because sequence encoding the anchoring structured domains is still present in Δ*FG nup* constructs.

The specific combinations of *FG NUP* or Δ*FG nup* genes cloned into a single expression plasmid was chosen based on the Nups, common physical association in NPC subcomplexes and/or NPC substructural localization (Figure 1 and Figure 2A). One set of plasmids harbored the three genes encoding the three FG Nups of the symmetric Nsp1 subcomplex: Nsp1, Nup49, and Nup57 (Grandi *et al.* 1995) (designated as *NSP1/NUP49/NUP57,* or *nsp1/nup49/nup57*Δ*FG* when lacking the FG domains). A second set contained genes encoding the two cytoplasmic-oriented FG Nups: Nup159 and Nup42 (designated as *C-WT*, or *C*Δ*FG* when lacking the FG domains), and a third, the three nuclear-oriented FG Nups: Nup1, Nup2, and Nup60 (designated as *N-WT*, or *N*Δ*FG* when lacking the FG domains).

**Figure 3:**
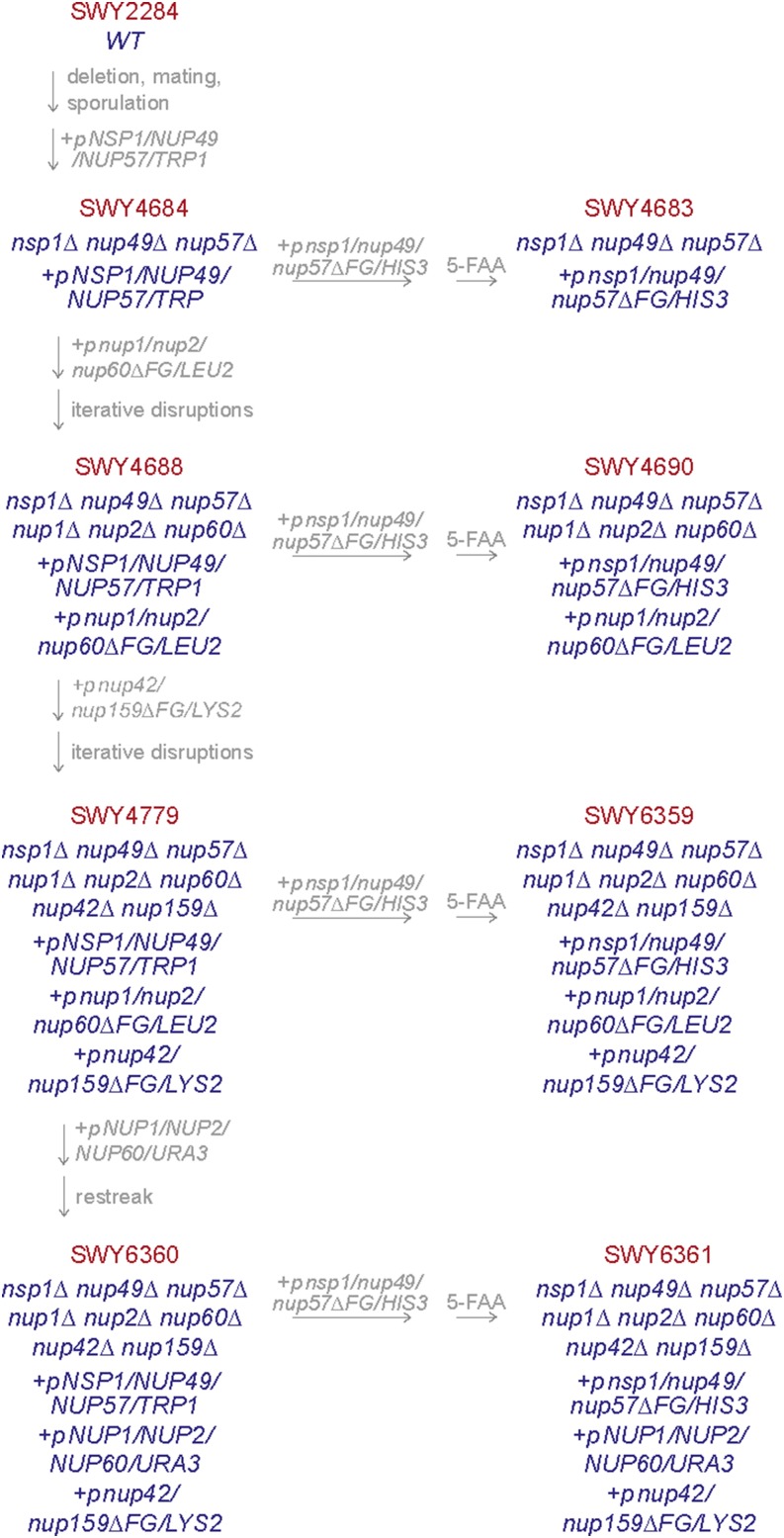
Construction history of Δ*FG* deletion strains. Beginning with a *WT* strain, *NSP1*, *NUP49*, and *NUP57* were individually deleted in the presence of a *WT NUP* vector. Strains were mated and sporulated to generate a triple null, and pSW3643 was transformed with counterselection of single gene-encoding plasmids to generate SWY4684. SWY4684 was transformed with pSW3547, and pSW3643 was counterselected on with the *TRP1* counterselective drug 5-FAA to generate SWY4683. SWY4684 was transformed with pSW3641, and *NUP1*, *NUP2*, and *NUP60* were deleted iteratively to generate SWY4688. SWY4688 was transformed with pSW3547, and pSW3643 was counterselected on 5-FAA to generate SWY4690. *LYS2* was deleted from SWY4688 with a floxed *SpHIS5* cassette, which was recombined. This strain was then transformed with pSW3646, and *NUP42* and *NUP159* were deleted iteratively to generate SWY4779. SWY4779 was transformed with pSW3547, and pSW3643 was counterselected on 5-FAA to generate SWY6359. SWY4779 was transformed with pSW3642, and colonies with spontaneous loss of *LEU2* were selected to generate SWY6360. SWY6360 was transformed with pSW3547, and pSW3643 was counterselected on 5-FAA to generate SWY6361. Additional strain and plasmid information is described in Table 1 and Table 2.

**Figure 4:**
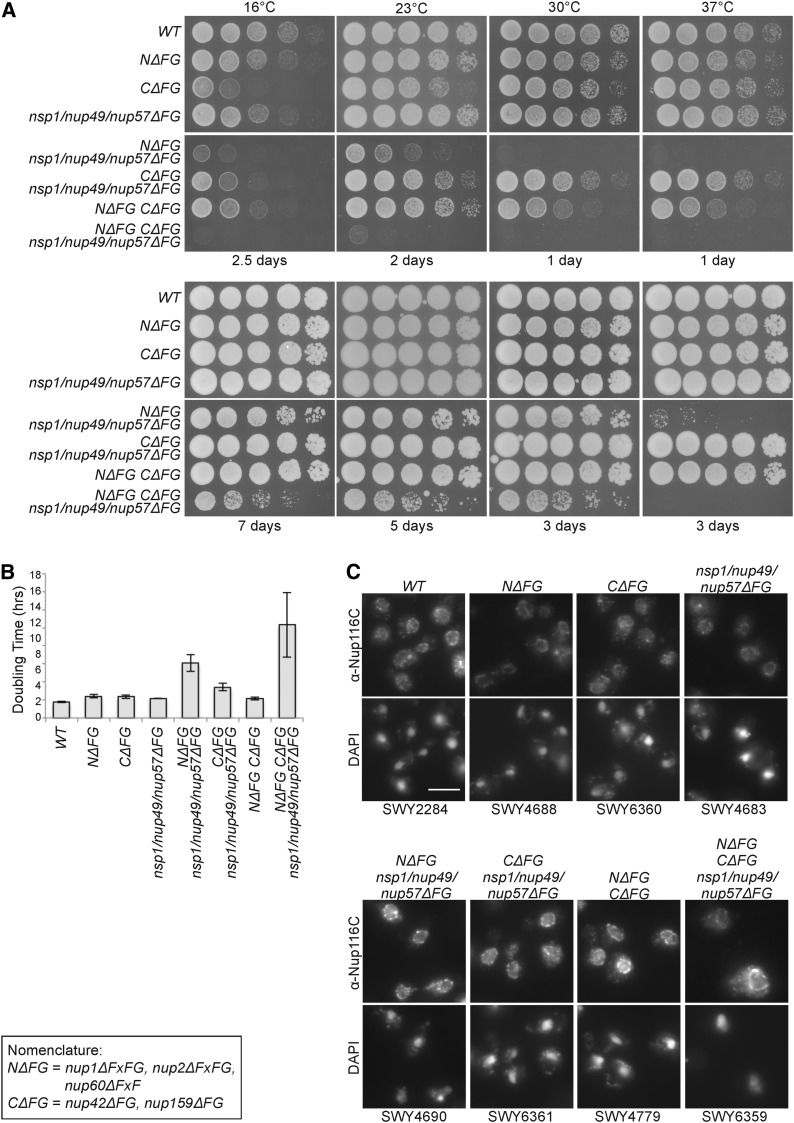
(A) Growth analysis of Δ*FG* strains at different temperatures. Yeast strains were grown at 23° to midlog phase and five-fold serially diluted on YPD plates for growth at the indicated temperature for 1–7 days. (B) Liquid growth analysis of Δ*FG* strains. Yeast strains were grown at 23° to early log phase, and OD_600_ was analyzed to determine doubling times. Error bars indicate standard deviation from three replicates. (C) Nup116 is properly assembled into NPCs of Δ*FG* strains. Indicated strains were grown at 23° to midlog phase and processed for indirect immunofluorescence microscopy using the anti-(α)-Nup116-CTD antibodies. DAPI staining marks the nucleus. *N*Δ*FG C*Δ*FG nsp1/nup49/nup57*Δ*FG* was scaled independently due to increased cellular autofluorescence. Scale bar, 5 μm.

By classic mating and sporulation, we first generated a triple deletion strain in which the endogenous chromosomal locus encoding each of the Nsp1-Nup49-Nup57 complex members was deleted in the presence of single *WT NUP* plasmids. The individual plasmids were then exchanged for a *NSP1/NUP49/NUP57* plasmid in the *nsp1*Δ *nup49*Δ *nup57*Δ triple mutant, which was subsequently exchanged for an *nsp1/nup49/nup57*Δ*FG* plasmid (Figure 3 and Table 1). We analyzed growth of the resulting *nsp1/nup49/nup57*Δ*FG* mutant by serially diluting equal numbers of cells onto rich media and growing the cells at the indicated temperatures (Figure 4A). The *nsp1/nup49/nup57*Δ*FG* mutant with the simultaneous deletion of all three of the FG domains in the Nsp1 complex was viable with no noted growth defects at the temperatures tested. This result was consistent with previous genetic analysis of the genes encoding this complex (Fabre *et al.* 1995), indicating that the reported lethality with the Cre-*loxP* approach was likely due to tag-specific effects (Strawn *et al.* 2004).

**Table 1:** Strain table

Strain	Description		Source
SWY2284	*MAT*α *trp1-1*, *ura3-1 his3-11,15*, *LYS2*, *leu2-3,112*		(Strawn *et al.* 2004)
SWY4684	*nsp1*::*KAN^R^ nup49*::*loxP nup57*::*loxP* *MAT*α *trp1-1*, *ura3-1 his3-11,15*, *LYS2*, *leu2-3,112* *pSW3554*		This study
SWY4683	*nsp1*::*KAN^R^ nup49*::*loxP nup57*::*loxP* *MAT*α *trp1-1*, *ura3-1 his3-11,15*, *LYS2*, *leu2-3,112* *pSW3547*		This study
SWY4688	*nsp1*::*KAN^R^ nup49*::*loxP nup57*::*loxP nup1*::*loxP nup2*::*loxP nup60*::*loxP* *MAT*α *trp1-1*, *ura3-1 his3-11,15*, *LYS2*, *leu2-3,112* *pSW3643 pSW3641*		This study
SWY4690	*nsp1*::*KAN^R^ nup49*::*loxP nup57*::*loxP nup1*::*loxP nup2*::*loxP nup60*::*loxP* *MAT*α *trp1-1*, *ura3-1 his3-11,15*, *LYS2*, *leu2-3,112* *pSW3547 pSW3641*		This study
SWY4779	*nsp1*::*KAN^R^ nup49*::*loxP nup57*::*loxP nup1*::*loxP nup2*::*loxP nup60*::*loxP nup42*::*loxP nup159*::*loxP* *MAT*α *trp1-1*, *ura3-1 his3-11,15*, *lys2*::*loxP*, *leu2-3,112* *pSW3643 pSW3641 pSW3636*		This study
SWY6359	*nsp1*::*KAN^R^ nup49*::*loxP nup57*::*loxP nup1*::*loxP nup2*::*loxP nup60*::*loxP nup42*::*loxP nup159*::*loxP* *MAT*α *trp1-1*, *ura3-1 his3-11,15*, *lys2*::*loxP*, *leu2-3,112* *pSW3547 pSW3641 pSW3636*		This study
SWY6360	*nsp1*::*KAN^R^ nup49*::*loxP nup57*::*loxP nup1*::*loxP nup2*::*loxP nup60*::*loxP nup42*::*loxP nup159*::*loxP* *MAT*α *trp1-1*, *ura3-1 his3-11,15*, *lys2*::*loxP*, *leu2-3,112* *pSW3643 pSW3642 pSW3636*		This study
SWY6361	*nsp1*::*KAN^R^ nup49*::*loxP nup57*::*loxP nup1*::*loxP nup2*::*loxP nup60*::*loxP nup42*::*loxP nup159*::*loxP* *MAT*α *trp1-1*, *ura3-1 his3-11,15*, *lys2*::*loxP*, *leu2-3,112* *pSW3647 pSW3642 pSW3636*		This study

Using the *nsp1*Δ *nup49*Δ *nup57*Δ triple mutant as a starting point, the sequences encoding the FG domains on the nuclear and cytoplasmic face of the NPC were subsequently deleted (Figure 3 and Table 1). Because higher order gene deletions are difficult to generate and track by mating and sporulation, we adopted an iterative approach in haploid strains where the endogenous *FG NUP* gene was deleted by a floxed *SpHIS5* cassette followed by recombination of the *SpHIS5* sequence by expression of Cre recombinase (Figure 2B). This approach permitted deletion of multiple genes within one strain without losing availability of auxotrophic markers. In order to accommodate available markers, the asymmetric *NUP* genes were deleted in the presence of *N*Δ*FG* and *C*Δ*FG* plasmids with *NSP1/NUP49/NUP57* covering the *nsp1*Δ *nup49*Δ *nup57*Δ deletions (Figure 3). We reasoned that this approach would not select for off-target effects because we previously observed that absence of all five asymmetric FG domains results in minimal growth defects (Terry and Wente 2007). Once the nuclear and cytoplasmic FG Nups were deleted, the WT *NSP1/NUP49/NUP57* plasmid was exchanged for the *nsp1/nup49/nup57*Δ*FG* plasmid.

By serial dilution and analysis of growth on YPD, we observed that absence of FG domains from both the Nsp1-Nup49-Nup57 subcomplex and the nuclear face had growth defects at all temperatures tested (*N*Δ*FG nsp1/nup49/nup57*Δ*FG*). In contrast, the absence of the FG domains both the Nsp1-Nup49-Nup57 subcomplex and the cytoplasmic face resulted in only mildly impacted growth (*C*Δ*FG nsp1/nup49/nup57*Δ*FG*) (Figure 4A). Deletion of all asymmetric FG domains in combination with *nsp1/nup49/nup57*Δ*FG* (*N*Δ*FG C*Δ*FG nsp1/nup49/nup57*Δ*FG*) resulted in a viable strain with drastic growth defects (Figure 4A). To quantitatively analyze growth of all strains, liquid culture growth analysis was conducted at 23° (Figure 4B). Whereas most strains had doubling times of ∼2 to 3 hr, *N*Δ*FG nsp1/nup49/nup57*Δ*FG* had a doubling time of 6.1 hr; *C*Δ*FG nsp1/nup49/nup57*Δ*FG*, 3.4 hr; and *N*Δ*FG C*Δ*FG nsp1/nup49/nup57*Δ*FG* 10.3 hr.

To assess whether NPCs are assembled in these Δ*FG* strains, indirect immunofluorescence microscopy was performed using an antibody raised against the carboxy-terminal (non FG) domain of Nup116. Nup116 is an FG Nup that localizes to cytoplasmic foci when NPC assembly is perturbed (Ryan and Wente 2002), and the vertebrate ortholog, Nup98, associates with the nuclear envelope only after scaffold Nups are recruited following mitosis (Dultz *et al.* 2008). Therefore, Nup116 localization to the NE rim is a marker for proper NPC assembly. In all the Δ*FG* strains tested, anti-Nup116 signal was located at the nuclear rim surrounding the nuclear DAPI signal, suggesting that NPC assembly was not notably altered in the mutants (Figure 4C).

The *N*Δ*FG C*Δ*FG nsp1/nup49/nup57*Δ*FG* strain results in a GLFG-only NPC: the GLFG domains of Nup100, Nup116, and Nup145 (paralogous to each other and orthologous to vertebrate Nup98; Ryan and Wente 2000) are the only FG domains remaining. The other two GLFG domains in Nup49 and Nup57 are absent. Considering previous reports that GLFG domains are required for the formation of the NPC permeability barrier (Hulsmann *et al.* 2012; Lord *et al.* 2015), and that modification of GLFG Nups relaxes the barrier *in vitro* (Labokha *et al.* 2013), this strain will be of interest for subsequent studies of NPC transport capacity and nuclear permeability. We have generated plasmids encoding Nup100, Nup116, and Nup145 and FG deletions (Table 2) for use in such analysis.

**Table 2:** Plasmid table

Vector	Name in Text	Description	Residues Deleted	Plasmid Backbone*^a^*	Auxotrophic Marker	ΔFG Restriction Site	Residues Added	Source
pSW222		*NSP1*		pRS315	*LEU2*			This study
pSW3428		*nsp1*Δ*FxFG*	179–591	pRS315	*LEU2*	*Nhe*I	Ala Ser	This study
pSW3524		*nsp1*Δ*FG*Δ*-FxFG*	3–591	pRS314	*TRP1*	*Spe*I	Thr Ser	This study
pSW3444		*NUP49*		pRS315	*LEU2*			This study
pSW3513		*NUP49*		pRS314	*TRP1*			This study
pSW3548		*NUP49*		pRS313	*HIS3*			This study
pSW3549		*nup49*Δ*GLFG*	2–223	pRS314	*TRP1*	*Spe*I	Thr Ser	This study
pSW3431		*NUP57*		pRS314	*TRP1*			This study
pSW3512		*NUP57*		pRS316	*URA3*			This study
pSW3550		*nup57*Δ*GLFG*	2–236	pRS314	*TRP1*	*Nhe*I	Ala Ser	This study
pSW3521		*NSP1*, *NUP57*		pRS316	*URA3*			This study
pSW3554		*NSP1*, *NUP49*, *NUP57*		pRS316	*URA3*			This study
pSW3555		*NSP1*, *NUP49*, *NUP57*		pRS313	*HIS3*			This study
pSW3643	*NSP1/NUP-49/NUP57*	*NSP1*, *NUP49*, *NUP57*		pRS314	*TRP1*			This study
pSW3551		*nup49*Δ*GLFG*, *nup57*Δ*GLFG*		pRS313	*HIS3*			This study
pSW3552		*nsp1*Δ*FG*Δ*-FxFG*, *nup57*Δ*GLFG*		pRS313	*HIS3*			This study
pSW3553		*nsp1*Δ*FG*Δ*-FxFG*, *nup49*Δ*GLFG*		pRS313	*HIS3*			This study
pSW3644		*nsp1*Δ*FG*Δ*-FxFG*, *nup49*Δ*GLFG*, *nup57*Δ*GLFG*		pRS315	*LEU2*			This study
pSW3547	*nsp1/nup49/* *nup57*Δ*FG*	*nsp1*Δ*FG*Δ*-FxFG*, *nup49*Δ*GLFG*, *nup57*Δ*GLFG*		pRS313	*HIS3*			This study
pSW812		*NUP1*		pRS315	*LEU2*			This study
pSW3634		*NUP1*		pRS314	*TRP1*			This study
pSW3637		*nup1*Δ*FxFG*	384–888	pRS315	*LEU2*	*Avr*II	Pro Arg	This study
pSW3635		*NUP2*		pRS314	*TRP1*			This study
pSW3638		*nup2*Δ*FxFG*	189–527	pRS314	*TRP1*	*Avr*II	Pro Arg	This study
pSW3636		*NUP60*		pRS314	*TRP1*			This study
pSW3639		*nup60*Δ*FxF*	397–512	pRS314	*TRP1*	*Avr*II	Pro Arg	This study
pSW3640		*NUP1 NUP2 NUP60*		pRS314	*TRP1*			This study
pSW3642	*N-WT*	*NUP1 NUP2 NUP60*		pRS316	*URA3*			This study
pSW3641	*N*Δ*FG*	*nup1*Δ*FxFG nup2*Δ*FxFG nup60*Δ*FxF*		pRS315	*LEU2*			This study
pSW3801		*NUP42*		pRS315	*LEU2*			This study
pSW3802		*NUP42*		pRS314	*TRP1*			(Adams *et al.* 2014)
pSW3645		*nup42*Δ*FG*	4–364	pRS315	*LEU2*	*Xho*I	Leu Glu	This study
pSW3448		*nup42*Δ*FG*	4–364	pRS317	*LYS2*	*Xho*I	Leu Glu	This study
pSW3657		*nup42*Δ*FG*	4–364	pRS314	*TRP1*	*Xho*I	Leu Glu	(Adams *et al.* 2014)
pSW3647		*NUP159*		pRS314	*TRP1*			(Adams *et al.* 2014)
pSW3648		*nup159*Δ*FG*	464–876	pRS314	*TRP1*	*Avr*II	Pro Arg	(Adams *et al.* 2014)
pSW3646	*C*Δ*FG*	*nup42*Δ*FG nup159*Δ*FG*		pRS317	*LYS2*			This study
pSW3500		*NUP100*		pRS313	*HIS3*			This study
pSW3501		*NUP100*		pRS314	*TRP1*			This study
pSW3502		*nup100*Δ*GLFG*	2–570	pRS313	*HIS3*	*SpeI*	Thr Ser	This study
pSW3503		*nup100*Δ*GLFG*	2–570	pRS314	*TRP1*	*SpeI*	Thr Ser	This study
pSW3504		*NUP116*		pRS313	*HIS3*			This study
pSW3506		*NUP145*		pRS314	*TRP1*			This study
pSW3656		*nup145*Δ*GLFG*	10–209	pRS314	*TRP1*	*NheI*	Ala Ser	This study

a These plasmids contains bacterial resistance (*AMP^R^*) and high copy replication (*ori*) sequences, yeast centromeric (*CEN6*) and replication (*ARSH4*) sequences, and the indicated yeast auxotrophic marker (Siskorski and Hieter 1989).

The plasmid-based expression of Δ*FG nups* in chromosomal null strains as presented here provides a straightforward way to introduce new sequences, mutations, or deletions into *nup* genes for analysis of FG Nup function *in vivo*. We previously assessed FG domain functional complementation using plasmid-based expression of Δ*FG nups* and *swapped FG (SFG) nups* (Iovine *et al.* 1995; Adams *et al.* 2014; Lord *et al.* 2015). The “swapped” strategy involves replacing the endogenous FG domain with that of another Nup. These studies revealed that FG domains of different Nups have inherently distinct function *in vivo*, because only select FG domains could functionally replace those tested. It is likely that sequence differences underlie distinct functionality. Indeed, individual domains from different FG Nups have distinct *in vitro* biochemical and biophysical characteristics (Lim *et al.* 2007; Yamada *et al.* 2010; Labokha *et al.* 2013). The genetic tools generated in this report will allow future investigations to conduct highly detailed tests of what sequences contribute to specialized function during transport and what biophysical and biochemical properties of FG domains contribute to the NPC permeability barrier and selectivity mechanism.

## Materials and Methods

### Yeast strains and growth

Table 1 lists the yeast strains generated in this study. Yeast genetic methods were conducted according to standard procedures (Sherman *et al.* 1986). Yeast strains were grown in either YPD (2% peptone, 2% dextrose, 1% yeast extract) or selective minimal media lacking appropriate amino acids and supplemented with 2% dextrose and 5-fluoroorotic acid (5-FOA; United States Biological) at 1.0 mg/mL or 2-amino-5-fluorobenzoic acid (5-FAA; Sigma-Aldrich) at 0.5 mg/mL as needed. For liquid culture analysis, strains were grown to early log phase (OD_600_ ∼0.15) at 23°, with OD_600_ measurements taken every 2 hr and normalized to time = 0.

### Plasmid construction

Table 2 lists the plasmid generated in this study. Plasmid cloning was performed according to standard molecular biology strategies, and Δ*FG* plasmids were generated by amplifying a wild type *NUP* plasmid to replace the FG domain with a unique restriction site (Figure 2A). Most FG domains were replaced with the restriction sites *Avr*II, *Nhe*I, and *Spe*I to generate compatible cohesive ends (with the exception of *Xho*I for *nup42*Δ*FG*). FG domain boundaries were based on Strawn *et al.* (2004), and indicated in Table 2. Immunoblotting confirmed loss of FxFG and GLFG domains in strains transformed with Δ*FG* plasmids (data not shown).

### Immunofluorescence

Yeast strains were grown to midlog phase (OD_600_ ∼0.5) in YPD medium at 23°, processed and labeled as in Ho *et al.* (2000). Briefly, samples were incubated with anti-Nup116-CTD rabbit antibodies (WU600, Iovine *et al.* 1995) overnight at 4°. Bound primary antibodies were detected with Alexa Flour 488-conjugated goat anti-rabbit IgG (1:200, Molecular Probes) and samples were stained with 0.1 mg/mL DAPI. Wide-field images were acquired using a microscope (BX50; Olympus) equipped with a motorized stage (Model 999000, Ludl), Olympus 100× NA1.3 UPlanF1 objective, and digital charge coupled device camera (Orca-R2; Hamamatsu). Images were processed with ImageJ (NIH).

### Data availability

Strains and plasmids are available upon request. Table 1 contains genotypes for each individual strain. Table 2 contains information for each plasmid.

## References

[bib1] Adams R. L. Wente S. R., 2013 Uncovering nuclear pore complexity with innovation. Cell 152: 1218–1221. 23498931 10.1016/j.cell.2013.02.042 PMC3672239

[bib2] Adams R. L. Terry L. J. Wente S. R., 2014 Nucleoporin FG domains facilitate mRNP remodeling at the cytoplasmic face of the nuclear pore complex. Genetics 197: 1213–1224. 24931410 10.1534/genetics.114.164012 PMC4125395

[bib3] Alber F. Dokudovskaya S. Veenhoff L. M. Zhang W. Kipper J., 2007 The molecular architecture of the nuclear pore complex. Nature 450: 695–701. 18046406 10.1038/nature06405

[bib4] Chug H. Trakhanov S. Hulsmann B. B. Pleiner T. Gorlich D., 2015 Crystal structure of the metazoan Nup62•Nup58•Nup54 nucleoporin complex. Science. 350: 106–110 26292704 10.1126/science.aac7420

[bib5] Davis L. I. Fink G. R., 1990 The NUP1 gene encodes an essential component of the yeast nuclear pore complex. Cell 61: 965–978. 2190694 10.1016/0092-8674(90)90062-j

[bib6] Del Priore V. Heath C. Snay C. MacMillan A. Gorsch L., 1997 A structure/function analysis of Rat7p/Nup159p, an essential nucleoporin of *Saccharomyces cerevisiae*. J. Cell Sci. 110(Pt 23): 2987–2999. 9359887 10.1242/jcs.110.23.2987

[bib7] Dultz E. Zanin E. Wurzenberger C. Braun M. Rabut G., 2008 Systematic kinetic analysis of mitotic dis- and reassembly of the nuclear pore in living cells. J. Cell Biol. 180: 857–865. 18316408 10.1083/jcb.200707026 PMC2265396

[bib8] Fabre E. Schlaich N. L. Hurt E. C., 1995 Nucleocytoplasmic trafficking: what role for repeated motifs in nucleoporins? Cold Spring Harb. Symp. Quant. Biol. 60: 677–685. 8824442 10.1101/sqb.1995.060.01.073

[bib9] Field M. C. Koreny L. Rout M. P., 2014 Enriching the pore: splendid complexity from humble origins. Traffic 15: 141–156. 24279500 10.1111/tra.12141 PMC3906644

[bib10] Grandi P. Schlaich N. Tekotte H. Hurt E. C., 1995 Functional interaction of Nic96p with a core nucleoporin complex consisting of Nsp1p, Nup49p and a novel protein Nup57p. EMBO J. 14: 76–87. 7828598 10.1002/j.1460-2075.1995.tb06977.x PMC398054

[bib11] Ho A. K. Shen T. X. Ryan K. J. Kiseleva E. Levy M. A., 2000 Assembly and preferential localization of Nup116p on the cytoplasmic face of the nuclear pore complex by interaction with Nup82p. Mol. Cell. Biol. 20: 5736–5748. 10891509 10.1128/mcb.20.15.5736-5748.2000 PMC86051

[bib12] Hulsmann B. B. Labokha A. A. Gorlich D., 2012 The permeability of reconstituted nuclear pores provides direct evidence for the selective phase model. Cell 150: 738–751. 22901806 10.1016/j.cell.2012.07.019

[bib13] Hurt E. C., 1988 A novel nucleoskeletal-like protein located at the nuclear periphery is required for the life cycle of *Saccharomyces cerevisiae*. EMBO J. 7: 4323–4334. 3072197 10.1002/j.1460-2075.1988.tb03331.x PMC455148

[bib14] Iovine M. K. Watkins J. L. Wente S. R., 1995 The GLFG repetitive region of the nucleoporin Nup116p interacts with Kap95p, an essential yeast nuclear import factor. J. Cell Biol. 131: 1699–1713. 8557738 10.1083/jcb.131.6.1699 PMC2120653

[bib15] Kabachinski G. Schwartz T. U., 2015 The nuclear pore complex–structure and function at a glance. J. Cell Sci. 128: 423–429. 26046137 10.1242/jcs.083246 PMC4311126

[bib16] Labokha A. A. Gradmann S. Frey S. Hulsmann B. B. Urlaub H., 2013 Systematic analysis of barrier-forming FG hydrogels from *Xenopus* nuclear pore complexes. EMBO J. 32: 204–218. 23202855 10.1038/emboj.2012.302 PMC3553378

[bib17] Lim R. Y. Fahrenkrog B. Koser J. Schwarz-Herion K. Deng J., 2007 Nanomechanical basis of selective gating by the nuclear pore complex. Science 318: 640–643. 17916694 10.1126/science.1145980

[bib18] Lim R. Y. Huang B. Kapinos L. E., 2015 How to operate a nuclear pore complex by Kap-centric control. Nucleus . 10.1080/19491034.2015.1090061 PMC4915502 26338152

[bib19] Lord C. L. Timney B. L. Rout M. P. Wente S. R., 2015 Altering nuclear pore complex function impacts longevity and mitochondrial function in *S. cerevisiae*. J. Cell Biol. 208: 729–744. 25778920 10.1083/jcb.201412024 PMC4362458

[bib20] Nehrbass U. Kern H. Mutvei A. Horstmann H. Marshallsay B., 1990 NSP1: a yeast nuclear envelope protein localized at the nuclear pores exerts its essential function by its carboxy-terminal domain. Cell 61: 979–989. 2112428 10.1016/0092-8674(90)90063-k

[bib21] Raices M. D’Angelo M. A., 2012 Nuclear pore complex composition: a new regulator of tissue-specific and developmental functions. Nat. Rev. Mol. Cell Biol. 13: 687–699. 23090414 10.1038/nrm3461

[bib22] Rout M. P. Wente S. R., 1994 Pores for thought: nuclear pore complex proteins. Trends Cell Biol. 4: 357–365. 14731624 10.1016/0962-8924(94)90085-x

[bib23] Rout M. P. Aitchison J. D. Suprapto A. Hjertaas K. Zhao Y., 2000 The yeast nuclear pore complex: composition, architecture, and transport mechanism. J. Cell Biol. 148: 635–651. 10684247 10.1083/jcb.148.4.635 PMC2169373

[bib24] Ryan K. J. Wente S. R., 2000 The nuclear pore complex: a protein machine bridging the nucleus and cytoplasm. Curr. Opin. Cell Biol. 12: 361–371. 10801463 10.1016/s0955-0674(00)00101-0

[bib25] Ryan K. J. Wente S. R., 2002 Isolation and characterization of new *Saccharomyces cerevisiae* mutants perturbed in nuclear pore complex assembly. BMC Genet. 3: 17. 12215173 10.1186/1471-2156-3-17 PMC126250

[bib26] Sherman F. Fink G. R. Hicks J. B. Cold Spring Harbor Laboratory, 1986 Laboratory course manual for methods in yeast genetics, Cold Spring Harbor Laboratory, New York, N.Y.

[bib27] Siskorski R. S. Hieter P. H., 1989 A system of shuttle vectors and yeast host strains designed for efficient manipulation of DNA in *Saccharomyces cerevisiae*. Genetics 122: 19–27. 2659436 10.1093/genetics/122.1.19 PMC1203683

[bib28] Strawn L. A. Shen T. Shulga N. Goldfarb D. S. Wente S. R., 2004 Minimal nuclear pore complexes define FG repeat domains essential for transport. Nat. Cell Biol. 6: 197–206. 15039779 10.1038/ncb1097

[bib29] Stuwe T. Bley C. J. Thierbach K. Petrovic S. Schilbach S., 2015 Architecture of the fungal nuclear pore inner ring complex. Science 350: 56–64. 26316600 10.1126/science.aac9176 PMC4826903

[bib30] Terry L. J. Wente S. R., 2007 Nuclear mRNA export requires specific FG nucleoporins for translocation through the nuclear pore complex. J. Cell Biol. 178: 1121–1132. 17875746 10.1083/jcb.200704174 PMC2064648

[bib31] Terry L. J. Wente S. R., 2009 Flexible gates: dynamic topologies and functions for FG nucleoporins in nucleocytoplasmic transport. Eukaryot. Cell 8: 1814–1827. 19801417 10.1128/EC.00225-09 PMC2794212

[bib32] Wente S. R. Rout M. P. Blobel G., 1992 A new family of yeast nuclear pore complex proteins. J. Cell Biol. 119: 705–723. 1385442 10.1083/jcb.119.4.705 PMC2289698

[bib33] Yamada J. Phillips J. L. Patel S. Goldfien G. Calestagne-Morelli A., 2010 A bimodal distribution of two distinct categories of intrinsically disordered structures with separate functions in FG nucleoporins. Mol. Cell. Proteomics 9: 2205–2224. 20368288 10.1074/mcp.M000035-MCP201 PMC2953916

